# Earthquake and typhoon trigger unprecedented transient shifts in shallow hydrothermal vents biogeochemistry

**DOI:** 10.1038/s41598-019-53314-y

**Published:** 2019-11-15

**Authors:** Mario Lebrato, Yiming V. Wang, Li-Chun Tseng, Eric P. Achterberg, Xue-Gang Chen, Juan-Carlos Molinero, Karen Bremer, Ulrike Westernströer, Emanuel Söding, Hans-Uwe Dahms, Marie Küter, Verena Heinath, Janika Jöhnck, Kostas I. Konstantinou, Yiing J. Yang, Jiang-Shiou Hwang, Dieter Garbe-Schönberg

**Affiliations:** 1 Institute of Geosciences, Kiel University (CAU), Kiel, Germany; 2 Bazaruto Center for Scientific Studies (BCSS), Benguerra Island, Mozambique; 3 Max Planck Institute for the Science of Human History, Jena, Germany; 4 National Taiwan Ocean University, Keelung City, Taiwan; 5 GEOMAR Helmholtz Centre for Ocean Research Kiel, Kiel, Germany; 6 Ocean College, Zhejiang University, Zhoushan City, China; 7 Marine Biodiversity, Exploitation and Conservation (MARBEC), IRD/CNRS/IFREMER/University of Montpellier, Montpellier, France; 8 Kaohsiung Medical University, Kaohsiung, Taiwan; 9 National Central University, Taoyuan, Taiwan; 10 National Taiwan University, Taipei City, Taiwan; 11 Jacobs University Bremen gGmbH, Bremen, Germany

**Keywords:** Element cycles, Ecosystem ecology, Marine chemistry

## Abstract

Shallow hydrothermal vents are of pivotal relevance for ocean biogeochemical cycles, including seawater dissolved heavy metals and trace elements as well as the carbonate system balance. The Kueishan Tao (KST) stratovolcano off Taiwan is associated with numerous hydrothermal vents emitting warm sulfur-rich fluids at so-called White Vents (WV) and Yellow Vent (YV) that impact the surrounding seawater masses and habitats. The morphological and biogeochemical consequences caused by a M5.8 earthquake and a C5 typhoon (“Nepartak”) hitting KST (12^th^ May, and 2^nd^–10^th^ July, 2016) were studied within a 10-year time series (2009–2018) combining aerial drone imagery, technical diving, and hydrographic surveys. The catastrophic disturbances triggered landslides that reshaped the shoreline, burying the seabed and, as a consequence, native sulfur accretions that were abundant on the seafloor disappeared. A significant reduction in venting activity and fluid flow was observed at the high-temperature YV. Dissolved Inorganic Carbon (DIC) maxima in surrounding seawater reached 3000–5000 µmol kg^−1^, and Total Alkalinity (TA) drawdowns were below 1500–1000 µmol kg^−1^ lasting for one year. A strong decrease and, in some cases, depletion of dissolved elements (Cd, Ba, Tl, Pb, Fe, Cu, As) including Mg and Cl in seawater from shallow depths to the open ocean followed the disturbance, with a recovery of Mg and Cl to pre-disturbance concentrations in 2018. The WV and YV benthic megafauna exhibited mixed responses in their skeleton Mg:Ca and Sr:Ca ratios, not always following directions of seawater chemical changes. Over 70% of the organisms increased skeleton Mg:Ca ratio during rising DIC (higher CO_2_) despite decreasing seawater Mg:Ca ratios showing a high level of resilience. KST benthic organisms have historically co-existed with such events providing them ecological advantages under extreme conditions. The sudden and catastrophic changes observed at the KST site profoundly reshaped biogeochemical processes in shallow and offshore waters for one year, but they remained transient in nature, with a possible recovery of the system within two years.

## Introduction

Shallow hydrothermal vents (<200 m water depth^1^) are geological features in active volcanic areas releasing hot fluids into the overlying seawater. These fluids are loaded with dissolved heavy metals, and in many cases, significant amounts of CO_2_ and SO_2_ gases^2,3^. Shallow vents are of major importance with respect to trace metal and elemental fluxes into neritic and shelf water masses for oceanic biogeochemical cycles. Vents, in general, provide insights into geochemical mass balance and ocean chemistry serving as source and sink of elements^4^ and are considered to be the first sites where complex organic compounds were synthesized mediating the appearance of early life forms on Earth^5,6^. Fluid composition is governed by heat-driven geochemical reactions between seawater and subsurface rocks, phase separation and re-condensation processes, direct release from crystallizing magma, and if the vents are close to mainland by groundwater discharges and meteoric water^3,7^. All compartments surrounding a venting area – sub-/seafloor, seawater and nearby pelagic and benthic ecosystems – are strongly influenced by the vents fluid composition and activity^8^.

Shallow hydrothermal vents have been used for environmental investigations around the world^1,9,10^ to study questions related to other natural processes and human-made perturbations on coastal and deep-sea ecosystems such as ocean acidification (OA), toxicity of industrial discharges, and seabed mineral mining and dredging^11^. They are of great value for research as they feature high concentrations of toxic metals and encompass challenging scenarios, e.g., future high CO_2_ and temperature on Earth; both variables are projected to increase - with detrimental consequences for marine organisms and ecosystems^12,13^. Therefore, shallow vent fields provide a unique setting for studying ecological^14^ as well as benthic organisms’ biological adaptation to extreme geochemical environments that may reflect global change scenarios (“a window into the future”)^10,15^. Because they are often located along active tectonic plate margins^1^, they are frequently impacted by extreme geological events such as earthquakes with landslides and tsunamis, or underwater volcanic eruptions. Typhoon-induced landslides in tropical areas make things even worse. Compared to deep-sea hydrothermal vents, shallow vent sites facilitate time series studies as it is easier and more cost-effective to regularly obtain samples. Yet, to the best of our knowledge, no biogeochemical time series study to date has been undertaken at shallow vents targeting changes over time by combining different disciplines, namely chemical oceanography, geochemistry, ecology, aerial drone imagery, and technical diving.

**Figure 1:**
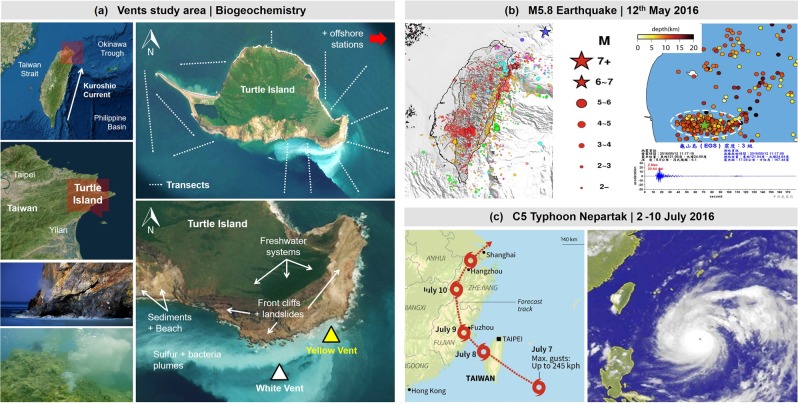
Turtle Island geographical situation and the catastrophic events that impacted the time-series work. (**a**) Shallow vents study area in Turtle Island off Taiwan, where work was conducted around the island, in the YV and in the WV. A summary of the project can be watched in Video S1. (**b)** Magnitude 5.8 earthquake that hit the south of Turtle Island on 12^th^ May 2016, triggering landslides, creating a new shoreline, and burying vents. Included are a map with earthquakes crust depth and the acceleration sensor data. The landslides that occurred during the earthquake can be watched in Video S2. (**c**) Category 5 typhoon Nepartak that hit Turtle Island and mainland Taiwan from 2^nd^ to 10^th^ of July 2016with large waves causing washout of sediments, particle/sediments resuspension and burial of vents. Detailed typhoon real-time data and photos can be found in Videos S3, S4, and details on rain, pressure, wind speed and significant wave height in Fig. S3. The final databases are deposited at the NOAA National Center for Environmental Information (NCEI) under Accession Number 0175781 in https://data.nodc.noaa.gov/cgi-bin/iso?id=gov.noaa.nodc:0175781 with DOI: 10.25921/6hy3-6d56. Aerial maps were obtained from Google, Google Earth, Images ©2018 CNES/Airbus,Data SIO, NOAA, U.S. Navy, NGA, GEBCO ©2018 Google. Earthquake data and mapping belong to co-author I. Konstantinou (unpublished). Typhoon imagery were obtained from the Central Weather Bureau (http://www.cwb.gov.tw/eng/). All imagery and maps were individually obtained, and the final figure was generated using Corel Draw X7 (Corel Corp.).

The Kueishan Tao (KST, 24.85°N, 121.95°E), also known as Turtle Island^10^, is a class T-hypsographic island and young stratovolcano (latest Holocene eruption 7000 years ago)^16,17^, located at a tectonic junction off northeast Taiwan and the southern end of the Okinawa Trough (Fig. 1; Videos S1, S2). The KST eastern face hosts a shallow-submarine hydrothermal venting system^1^ directly facing the Kuroshio Current (current speed = 0.36 to 2.02 m s^−1^) that hits the KST vent field^18^. The vents take the full strength of Pacific Ocean swells that are magnified by typhoons at peak season between May and October (Fig. 1; Videos S3, S4). Wind speed can vary from 0 up to 30 m s^−1^ during winter season, with strong waves that can reach 4 to 6 m and, in extreme cases 10 to 12 m during typhoons directly hitting the KST vent field (NTU1 and NTU2 buoys; Fig. S1). The year-round Taiwan weather brings copious rainfall that is magnified on typhoon season peaking at 200 to 300 mm month^−1^ in many areas including KST (the island has its own freshwater stream system and groundwater reservoirs) (Fig. S1). Seismic studies indicate continuous regional micro earthquakes (annual mean >400 events) (Fig. 1), with some being large enough to rupture volumes of crustal rocks^19^ forming open faults and fissures or, vice versa, closing pathways in the plumbing system. Earthquakes also trigger landslides from KST cliffs (Videos S1, S2). The vent field substratum is formed by rocky outcrops, volcanic debris and tophaceous sand and sulphur deposits which along with the shoreline and the seabed are continuously being eroded by typhoons, waves, and earthquakes.

The KST water masses’ carbonate chemistry and geochemistry is variable, with pH_total_ reaching extremely low (1.52 to 6) values, and wide dissolved heavy metal fluctuations^2,3^. More than 50 hydrothermal vents expelling whitish and yellowish fluids, thus called Yellow Vents (YV) and White Vents (WV) are distributed over an area of <1 km^2^ at depths of 6 to 30 m, with venting activity extending to 300 m depth (Fig. 1). The KST site is particularly suitable for this study because the shallow vents expel strong gas flows^2^ with fluids composed of >92% CO_2_ (via HCO_3_^−^) and additional SO_2_ degassing lowering the pH_total_ down to 2 in some areas (5–6 on average), with high H_2_S (up to 12%) but low HCl concentrations^2,3^. There are also elevated concentrations of seawater dissolved heavy metals. The vent fluids are warm with reported maximum boiling temperatures up to around 105 to 126 °C (YV) depending on water depth, making plumes positively buoyant and, thus, transporting dissolved components to the surface^2,3^. The high fluid ^3^He/^4^He ratios (>7), which are the highest in the west Pacific region^20^, suggest that the hydrothermal fluids react with components from the upper mantle and have only minor interaction with subducted sediments or dissolved atmospheric compounds like meteoric water. However, KST is currently undergoing decreased volcanic and venting activity which resulted in a gradual fluid compositional change coupled to wide seawater carbon chemistry variability^21^. The seawater in the vents area is full of chemosynthetic bacteria (epsilonproteobacteria and gammaproteobacteria) providing a conspicuous white colour (Video S1)^22^. Despite the extreme environment, opportunistic benthic organisms are abundant, but only two species of specialist crab (*Xenograpsus testudineus* and *Macromedaeus distinguendus*) inhabit and feed close to the YV^23^. Further apart in the WV area, corals and anemones thrive, increasing in density with distance from the vents in a peripheral zone where organisms have physiologically adapted to the chemistry^8^.

In 2016, a M5.8 earthquake and a C5 typhoon (“Nepartak”) hit KST within a few weeks apart (12^th^ May and 2^nd^–10^th^ July). Although typhoons and earthquakes are recurrent events at KST, they rarely occur in conjunction. Consequently, these events caused large scale landslides and many vents were buried. This major disturbance provided the unique opportunity to quantify catastrophic events’ transient impact on seawater biogeochemical processes around KST water masses and benthic ecosystems as part of a time series study, which has to the best of our knowledge rarely been done before. Here, we present results from a time-series study combining both biogeochemical measurements (2009–2018) and aerial drone photo/video data (1960–2017) for the KST shallow vents. Combining fluid and ambient seawater chemistry, chemical oceanography, aerial drone imagery, and benthic organisms’ skeleton elemental composition we provide a complete set of information to discuss the transient environmental consequences of these episodic extreme phenomena in the context of long-term variability of environmental parameters in both space and time. Because of the KST decreasing volcanic and venting activity as well as gas chemical composition over time we expect changes in dissolved seawater heavy metals as well as larger fluctuations in seawater carbonate chemistry. Also, owing to the earthquake and typhoon major disturbances, we expect sudden and pronounced shifts in seawater biogeochemistry that may in turn impact benthic megafaunal organisms’ skeleton elemental ratios. Our time-series approach captures, for the first time, how a major natural disturbance impacts biogeochemical transient processes at shallow vents that subsequently affect the surrounding habitats and shallow water masses including the open ocean.

## Results

**Figure 2:**
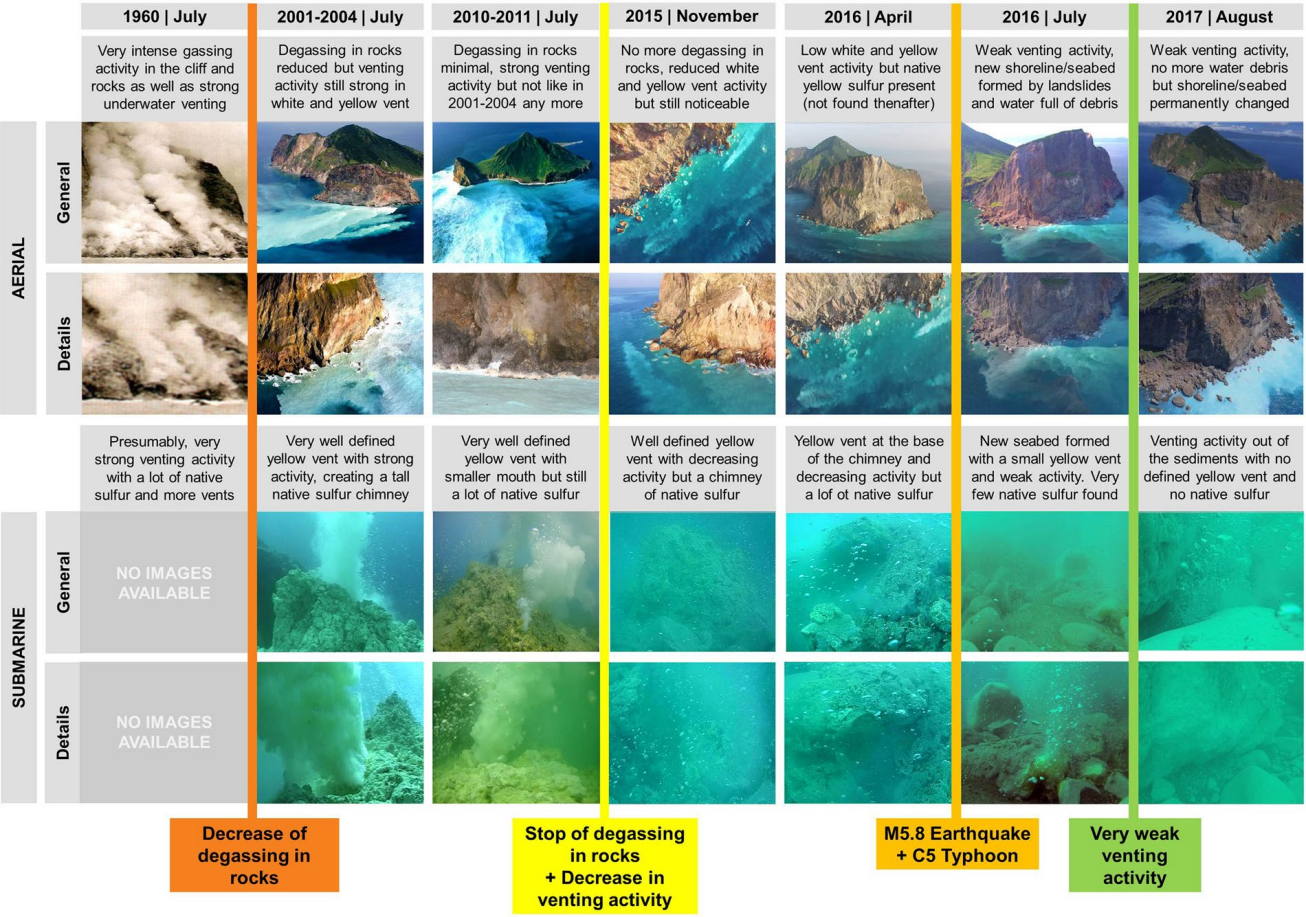
Aerial and submarine photo time-series of Turtle Island vents field changes during the Holocene (modern times). Detailed photographic work from 1960 to 2017 on Turtle Island vents field changes over time as a function of decreasing venting activity, degassing and a series of catastrophic events (M5.8 earthquake and C5 typhoon). Aerial photos were obtained using helicopters and drones (Dji Phantom 3, 4, and Mavic Pro), while submarine images were taken during dives using waterproof cameras (GoPro 3 and 4). More details on each time period can be found above the aerial and underwater photos. The colored bars and text indicate major historical changes that impacted the venting activity. For zoomed in images and details watch Video S1.

**Figure 3:**
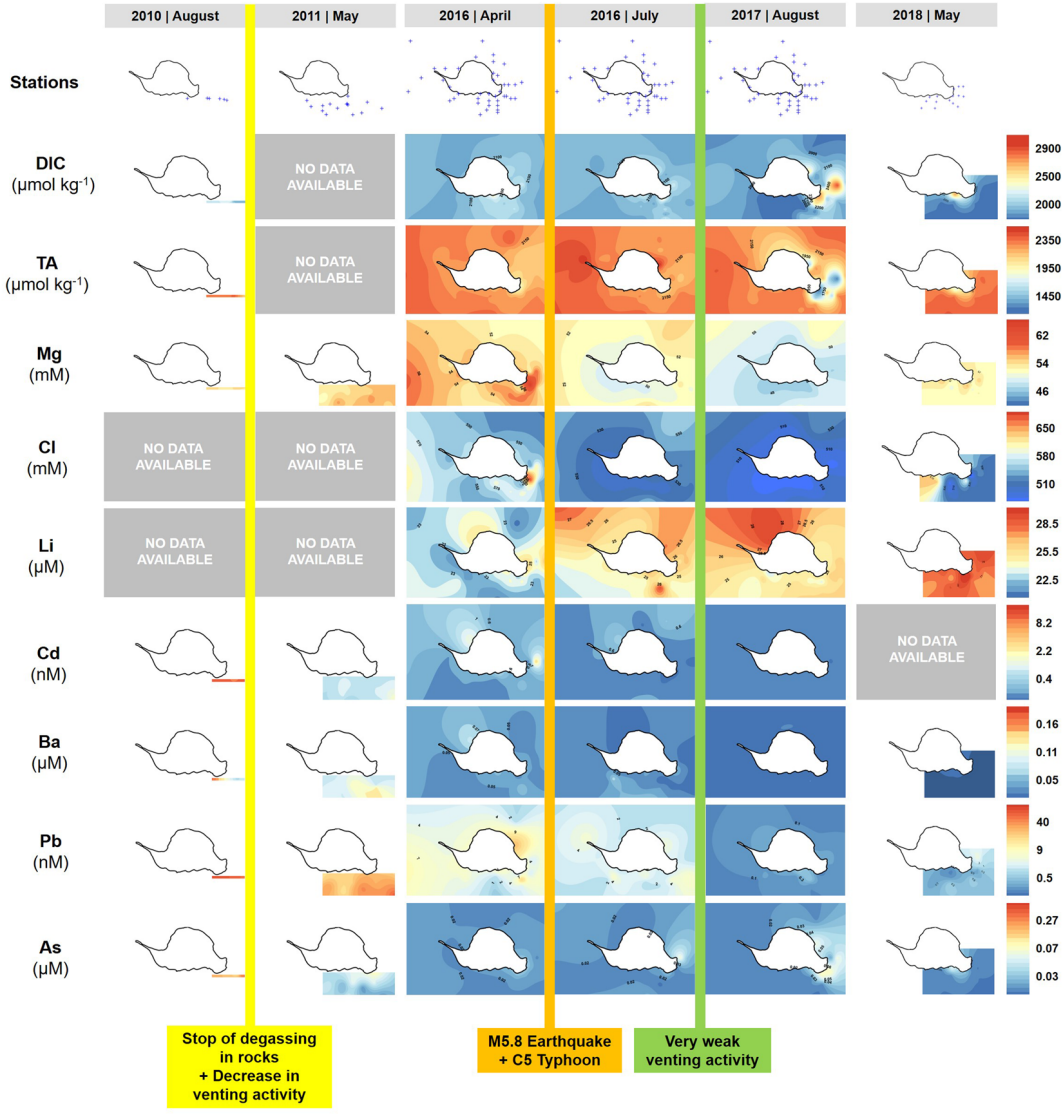
Seawater chemistry spatial distribution time-series before and after the catastrophic events. Seawater carbonate chemistry and selected dissolved elements (majors and minors) spatial distribution from 2010 to 2018 around Turtle Island and the vents, including offshore waters using gridded stations (all samples taken 1 m below the surface). Comparable seawater data before 2010 are not available, thus not included in the time-series, in contrast to Fig. 2 photos. The complete dataset of all chemistry, measurements, metadata, and seawater major/minor elements is included in Appendix S1.

In order to study changes in KST venting activity, we analysed aerial drone, underwater photo and video evidence in the same season over several periods from 1960 to 2017. We also included recurrent seawater spatial hydrographic surveys and YV mixing zone samples (environmental data, nutrients, carbonate chemistry and dissolved metals) taken from the same sampling stations from 2009 to 2018 (Figs 2, 3; Appendix 1). The final databases are deposited at the NOAA National Center for Environmental Information (NCEI) under Accession Number 0175781 in https://data.nodc.noaa.gov/cgi-bin/iso?id=gov.noaa.nodc:0175781 with 10.25921/6hy3-6d56.

The largest sampling effort occurred from 2015 to 2018 coinciding with the M5.8 earthquake and the C5 typhoon hitting KST within a few weeks apart (12^th^ May and 2^nd^–10^th^ July, 2016) (Fig. 1; Video S1). Aerial photos/videos time-series showed that the KST experienced a gradual decrease in degassing activity from 2001 to 2014, and then drastic changes (in degassing and landscaping) after the earthquake and typhoon (see Video S1 for details). The images from 1960 revealed intense degassing from on-shore fumaroles and solfataras on rocks/cliffs coupled to very strong submarine venting; unfortunately, no underwater images or seawater data exist from this period (Fig. 2). Until 2001–2004, degassing from the cliff rocks decreased but venting activity remained very high as evidenced from cloudy seawater around the vent field, being maintained until 2009–2011. From 2011 to 2015 degassing in rocks/cliffs ceased completely and venting activity decreased with a smaller YV chimney structure observed (Fig. 2). From 2015 to 2016 no major changes occurred until May and July 2016 when the M5.8 earthquake and C5 typhoon hit KST and triggered landslides and post-typhoon waves. This caused massive sediment washouts and seawater particle clouds that mainly originated from mass wasting (Fig. 2; Video S2). These catastrophic events buried the original shoreline and seabed under 1 to 3 m of sediment down to 6–8 m water depth, also creating brown particle plumes that extended with tides and current flow for hundreds of meters and settled down to 30 m. The old seabed and vent orifices (including sulfur accretions) were covered under the particle/debris-load and deposits making the underwater landscape unrecognizable (Fig. 2). The few vents found after the events were only weakly bubbling through the newly formed seabed. All native yellow sulfur which was very common before the events was potentially disintegrated and washed out by wave action and/ or completely buried, and none could be found thereafter on the seafloor. From 2016 to 2017 all sediments and particle plumes settled but the venting activity further decreased, with the YV now significantly reduced in size and no discernibly accretion of freshly formed native sulfur. In 2018, the venting activity increased slightly but yet no native sulfur accretions could be found.

**Figure 4:**
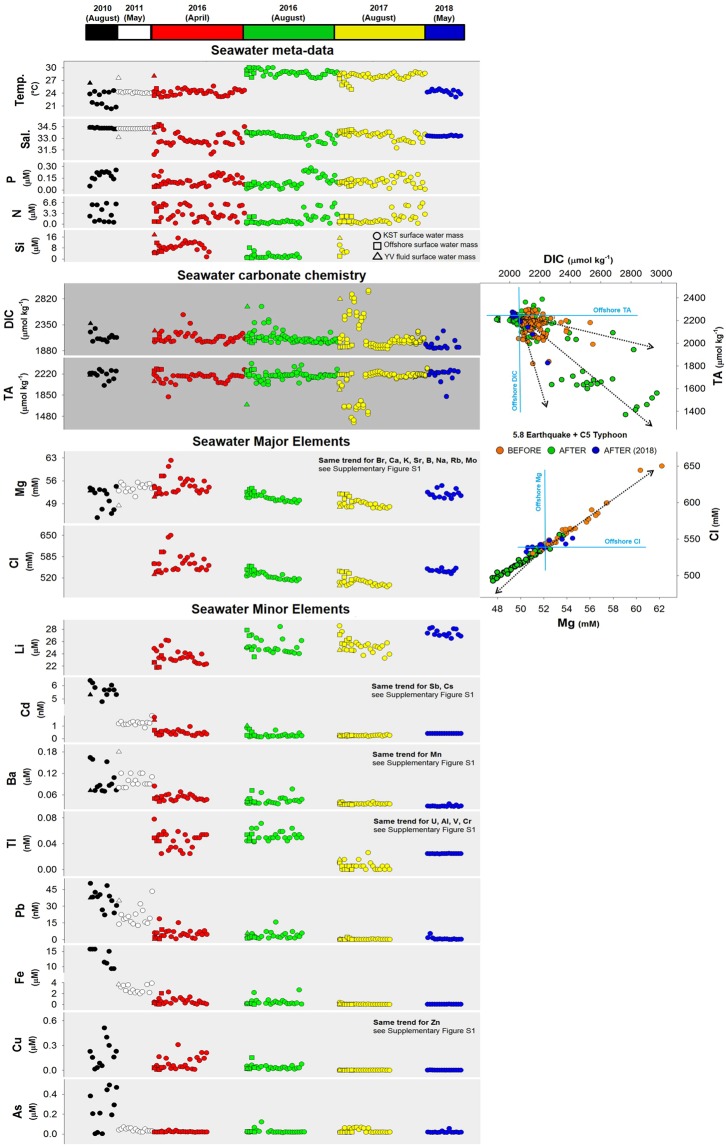
Seawater chemistry detailed time-series before and after the catastrophic events. Detailed spatial distribution of seawater carbonate chemistry and selected dissolved major/minor elements from 2010 to 2018 around Turtle Island and the vents, including offshore waters (square symbols) using gridded stations (all parameters measured 1 m below surface). Comparable seawater data before 2010 are not available, thus not included in the time-series, in contrast to the photos of Fig. 2. The complete dataset of all chemistry, measurements, metadata, and seawater major/minor elements is included in Appendix S1. Extended data can be found in Fig. S1.

**Figure 5:**
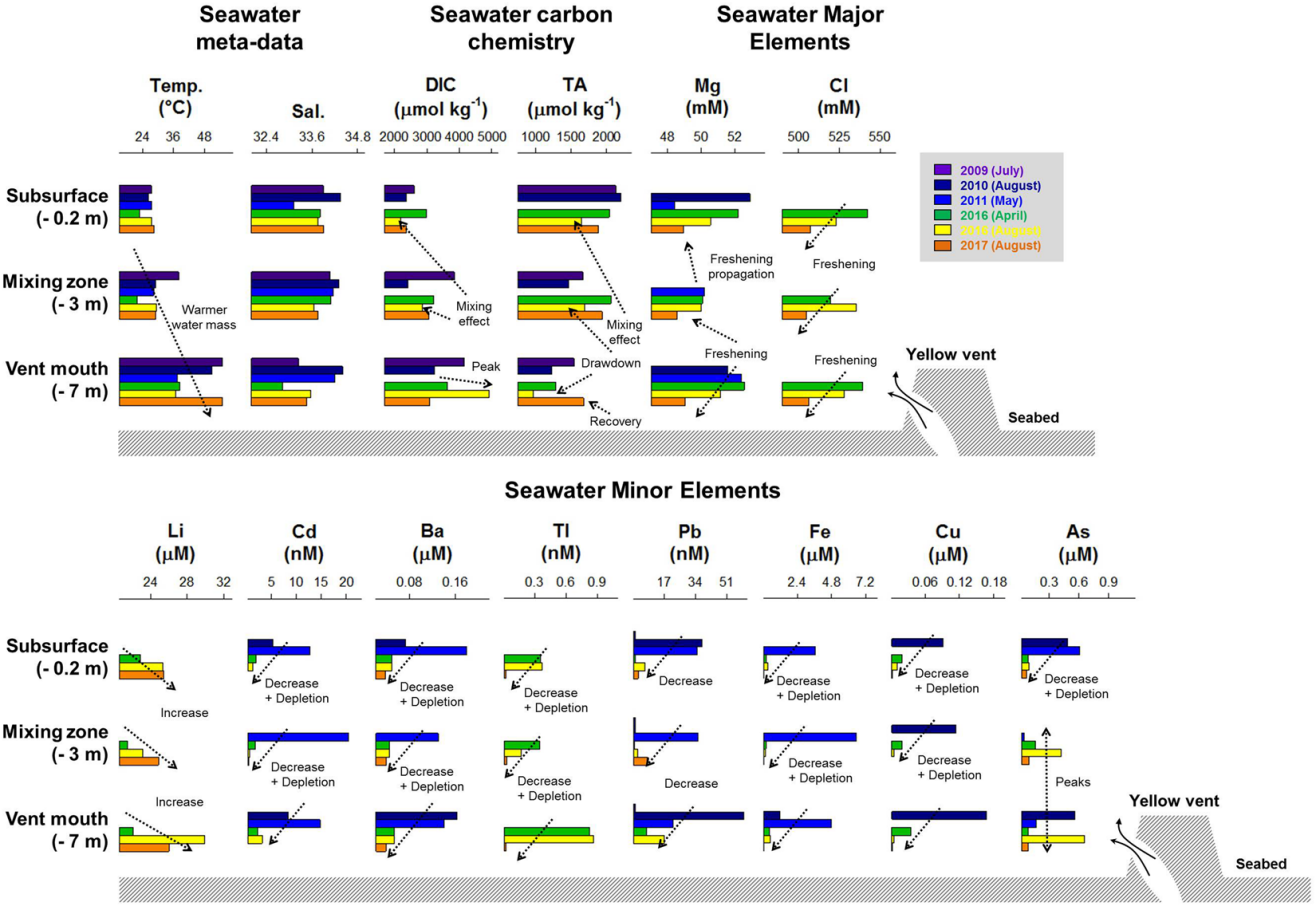
Yellow Vent (YV) vertical seawater chemistry and elemental composition time-series. Detailed vertical structure of the YV seawater carbonate chemistry and dissolved elements (majors and minors) from 2009 to 2017. For each parameter the full time-series data are presented in the same vertical plot to see individual changes over time. Comparable YV vertical seawater data before 2009 are not available, thus not included in the time-series, in contrast to the photos of Figs 1, 2. The complete dataset of all chemistry, measurements, metadata, and seawater major/minor elements is included in Appendix S1. Extended data can be found in Fig. S2.

To study temporal biogeochemical changes in the KST vent system, we measured environmental parameters (temperature, salinity, and nutrients – dissolved silicon (Si), dissolved inorganic nitrogen (DIN, N), dissolved inorganic phosphorous (DIP, P), seawater carbonate chemistry (TA and DIC), and dissolved major/minor metals along a sampling grid in the water masses around the island and the open ocean (Figs 3, 4), and vertically above the YVs mouth (Figs 4, 5) from 2009 to 2018, in particular from 2015 to 2018 (Appendix 1). We found three major patterns; (1) water masses composition, temperature and salinity remained relatively constant until 2016; (2) many parameters rapidly increased or decreased by several units after the earthquake and typhoon in both the shallow water masses and the open ocean; (3) major elements recovered in 2018 back to pre-disturbance levels (Fig. 4). Bioavailable nutrients (P, N, Si) were always variable over the studied time-span but, after the disturbance they were almost depleted to very low concentrations close to detection limits as a consequence of a phytoplankton bloom in the vicinity of the vent water masses (Fig. 4). Dissolved inorganic carbon (DIC) and total alkalinity (TA) remained relatively constant over time in the vent water masses and open ocean below 2350 µmol kg^−1^ and above 2100 µmol kg^−1^ until the disturbance. Afterwards, these parameters experienced extreme increases and drawdowns (up to 2800 and down to 1400 µmol kg^−1^, respectively) in the vent water masses, but the impact did not arrive to the open ocean. The peaks (>2350 µmol kg^−1^) and (<2000 µmol kg^−1^) in DIC and TA persisted until 2017 (Fig. 4). In 2018, DIC recovered to pre-disturbance levels below 2350 µmol kg^−1^, but TA still showed some drawdowns around 1850 µmol kg^−1^ (Fig. 4). Seawater major ions such as Mg and Cl remained mainly constant in the vent water masses and the open ocean around 55 mM and 585 mM, respectively, with seasonal maxima of 60–63 mM and 590–650 mM until 2016. But immediately after disturbance, Mg and Cl dramatically decreased to 48–55 mM and 520–580 mM (Fig. 4) both in the vent water masses and the open ocean. In 2018, both elements recovered to pre-disturbance concentrations but showing more scatter (Fig. 4). The same occurred for dissolved Br, Ca, K, Sr, B, Na, Rb, and Mo in the vent water masses and open ocean, but in 2018 not all elements (Cd, Ba, Tl, Pb, Fe, Cu, As, Cs, Al, Cr, Mn, Zn) recovered to pre-disturbance levels (Figs 4, S2,S3). Hydrothermal input of trace metals such as Cd, Ba, Tl, Pb, Fe, Cu and As steadily decreased until 2016, and immediately after the disturbance they were almost depleted below detection limits both in the vent water masses and the open ocean (Fig. 5). Similar depletion trends were observed for Sb, Cs, Mn, U, Al, V, Cr and Zn (Figs S2, S3). The only exception was dissolved Li that increased over time around the island with several maxima in the water masses from 24 µM to 26–28 µM, including 2018 (Fig. 4).

Vertical profiles above the YV showed the same trend of increasing concentrations towards the vent mouth as in the spatial surveys from 2009 to 2017 (Fig. 5) being a source especially for Al, Mn, Fe, and heavy metals Cd, Tl, Pb, Cu, etc. Temperature increased with depth towards the vent up to 50 °C in the vent mouth mixing zone, but salinity did not change indicating that fluids have near seawater salinity. A mixing and dilution effect on the seawater carbonate chemistry was observed from the YV mouth up to the surface. DIC peaked up to 5000 µmol kg^−1^ and TA showed drawdowns below 1000 µmol kg^−1^, both disclosing large variability. No nutrient samples were available from the YV. Temporally, seawater Mg and Cl showed a freshening effect after the events (Fig. 5), with the same decreasing trend and depletion for Br, Ca, K, Sr, B, Na, and Mo (Figs S2, S3). Seawater minor metals (Cd, Ba, Tl, Pb, Fe, and Cu) decreased and became almost depleted. Only the concentration of Li increased, and that of As showed high variability (Fig. 5). Similar trends were observed for Sb, Cs, Cr, Mn, and Zn (Figs S2, S3). Other metals such as U, Al and V were highly variable with no trend in the YV vertical distribution (Figs S2, S3).

**Figure 6:**
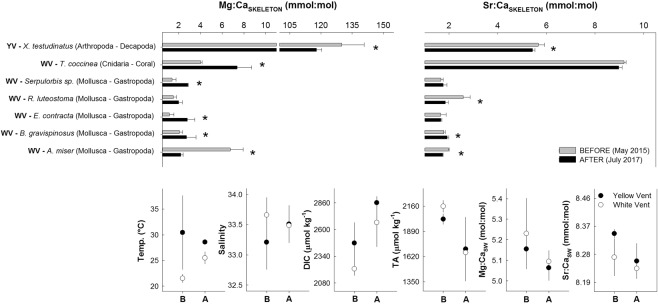
Benthic organisms’ skeleton calcite chemistry before and after the catastrophic events. Skeleton Mg:Ca and Sr:Ca ratios from the YV (YV) and WV (WV) benthic organisms collected before (grey bar) (May 2015) and after (black bar) (July 2017) the M5.8 earthquake and C5 typhoon. The *indicates t-test statistical significance for each paired comparison (see full results in Table S1). The *in situ* benthic seawater chemistry of the YV (YV, black circle) and WV (WV, white circle) associated with the organisms is also included before (**B**) (May 2015) and after (**A**) (July 2017) the events (see full data in Table S2).

The response of different megafaunal benthic organisms’ skeleton carbonate to the disturbance and subsequent seawater changes from the 2015 to 2017 surveys (before and after the extreme events) was variable. Roughly 70% of the species (*n* = 7) increased the skeleton Mg:Ca ratio after the extreme DIC increase (CO_2_ increase) despite decreasing seawater Mg:Ca ratios. Yet, the Arthropod *X. testudinatus* and the Mollusc *A. miser* decreased the skeleton Mg/Ca (Fig. 6; for statistical significance refer to Table S1, and for the full data to Table S2). As of Sr:Ca ratios, around 45% of the species (*n* = 7) showed an increase after the disturbance. The degree of change in skeleton Sr:Ca ratios was smaller than those in Mg:Ca ratios (Fig. 6).

## Discussion

The heat source for the current hydrothermal processes at Kueishan Tao (KST) is thought to be a small shallow magma chamber and/or a number of dykes and melt pockets beneath the volcano that are slowly solidifying and cooling unless a fresh injection of melt is reheating the system and eventually causing an eruption of the volcano with renewed degassing and hydrothermal activity. Hence, after an eruption, the heat flow and release of magmatic gases can be expected to die down slowly over time. Moreover, crystallization of hydrothermal minerals in the up-flow zone of a hydrothermal system may lead to clogging of fluid pathways (the plumbing system) and progressively reduced fluid flow. Tectonic activity causing faulting and triggering earthquakes may also have a direct effect on the plumbing system by changing or blocking fluid pathways. The relative volcanic hydrothermal and degassing activity over historic time can be estimated from on-site and aerial photography of on-shore fumaroles and solfataras and discoloration of the seawater around the island caused by sulfur-rich gases and fluids (Fig. 2). The KST hydrothermal system has been decreasing in activity with respect to major geophysical and biogeochemical processes for at least 60 years with a critical “dying down phase” over the last 20 years, and a sudden “near shutdown” after the 2016 M5.8 earthquake and C5 typhoon (Fig. 2). However, the “dying down” may have started right after the last eruption during the Holocene (7000 years ago), which triggered the shallow hydrothermal venting activity and degassing through both submarine vents and fumaroles and solfataras in the cliffs. It remains unclear if KST goes through volcanic cycles and peaks of activity that expand and contract the area of the venting field^24^, shifting the fluids’ chemical composition, and impacting on benthic organisms and ecosystems. If running-through-cycles is the case for KST then we witnessed the down cycle of the vent field’s activity over time, but this remains to be confirmed in the future to rule out the possibility of a progressive unidirectional shutdown and complete closure within a lifetime. However, the geological time scale is much longer and cannot easily be predicted.

KST seawater masses chemistry in the mixing zone and the open ocean are affected by two kinds of hydrothermal fluids. One is the Yellow Vent (YV) fluid with a strong fluid flow and recorded temperatures above 100 °C, and the other is the White Vents (WV) fluid having weaker fluid flow and lower recorded temperatures of up to 60 °C combined with strong degassing activity. The observed large decreases in dissolved Mg and Cl (Fig. 3) in ambient seawater after the disturbance events, and subsequent recovery in 2018, however, suggest further input sources such as meteoric water, coastal groundwater discharge, and island runoff fluxes (see below).

**Figure 7:**
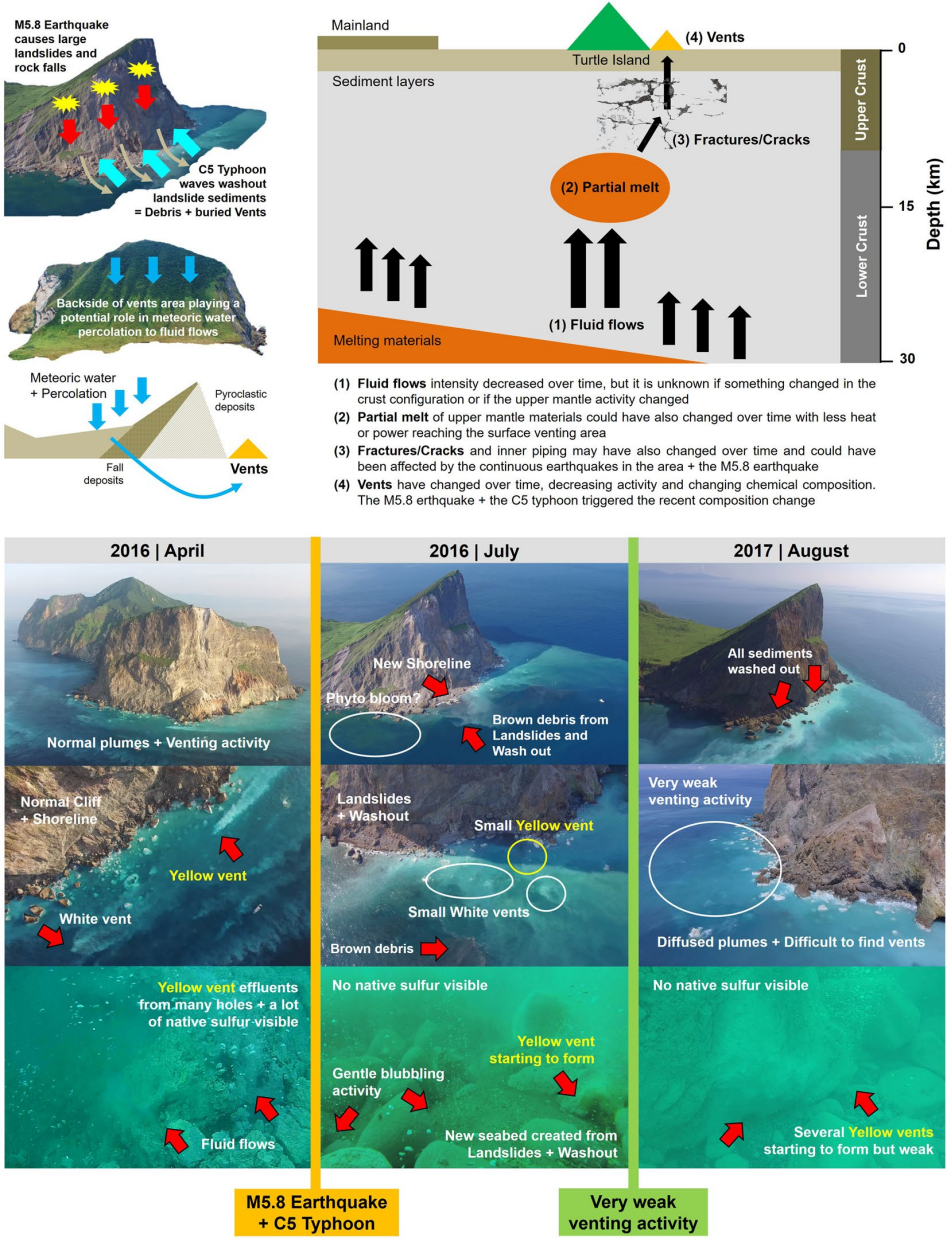
Identified processes that impacted Turtle Island vents geophysics and biogeochemistry during the Holocene (modern times). Detailed explanations of the processes believed to have impacted the vents over historical times with a focus on the recent M5.8 Earthquake and C5 Typhoon. Also included images with detailed descriptions of the major geophysical changes observed before and after the catastrophic events. For zoomed in images and details watch Video S1. All imagery and diagrams were individually produced, and the final figure was generated using Corel Draw X7 (Corel Corp.).

The vents fluids flowing into the mixing zone are affected by both upper mantle and crustal processes^20^, but possibly also by entrainment of groundwater or sub-lacustrine fluxes (percolation down to ground level^7,25^). However, ^3^He/^4^He ratios of >7 make such a contribution of surface water unlikely – at least in the past when those measurements had been made^20^. Samples from the mixing zone of ambient seawater mixed with vent fluids showed sudden shifts in seawater carbonate chemistry with peaking and drawdown activity of DIC and TA after the disturbance (Figs 3–5). This indicates higher volatilization of CO_2_ via magma degassing and subsequent solution as HCO_3_^−^, and additional sources of groundwater HCO_3_^−^ enrichment (also suggested by^2,3^ and quantified by^17^ using isotopes). The TA/DIC strong signal in the water masses of the mixing zone was seen again during 2017, either as a consequence of the 2016 events or owing to a fundamental change in the hydrothermal fluids per se (not sampled inside the vents). Yet, in 2018 DIC and TA recovered to pre-disturbance levels, indicating again changes in the system. The TA shifts were mostly caused by the earthquake-induced landslide particles and debris combined with the recurrent typhoon and wave’s sediment washouts forming particle-clouds with clay minerals as observed near the vents (Fig. 7; Videos S1–S3). The KST site was previously hypothesized to drawdown large TA amounts over time (near 4.5 × 10^7^ mol yr^−1^)^2,3^, but TA was never studied in detail. We suggest that the disturbance events magnified this TA drawdown flux, extending its intensity likely for several years, yet, eventually, TA drawdown intensity comes back to pre-disturbance levels. This TA-drawdown process as a consequence of ion-exchange and adsorption to clay particles has been described in river mouths and in some cold-warm water fronts^26^, but never before in relation to hydrothermal systems. With a lot of clay minerals and muddy plumes in the seawater, cation/anion exchanges occur between vent water and seawater that alter the TA, impacting on the TA drawdown flux^2,3^. These processes are similar to coastal wastewater and sewage discharges via rivers, or to industrial areas with disposals of heavy metals-bearing sludges that affect the near coastal environment^27,28^. However, KST being an open ocean setting facilitates the return to normality within 1 or 2 km of water masses, while in coastal areas, water bodies are either enclosed or with poor water mass exchange.

The decrease in salinity of water masses around KST was likely triggered by freshwater via meteoric or ground water entrainment around the island (Figs 4, 5)^17^. The original Mg and Cl values were slightly higher than normal seawater in the vent water masses and open ocean before the disturbance events, but showed depletions thereafter and a recovery after two years. The observed shifts in most major/minor elements with strong decrease and depletion after the disturbance are likely driven by dilution and freshening of seawater around KST water masses with an extension also to the open ocean in the vicinity. The recovery of Mg and Cl concentrations in 2018 back to pre-disturbance levels illustrates the time scale (two years) that is possibly needed by the entire KST system to settle after the earthquake and typhoon. It has been suggested that Cl can also fluctuate with variable input from magmatic volatiles and precipitates that are highly water soluble^2^.

Shallow vent fluid chemistry in island arc settings is dominated by degassing of magmatic volatiles and especially sulfur species creating strong acidity of “acid-sulfate” type fluids. These fluids do not react with the host rock as in deep-sea systems with convection cells and, consequently, do not lose the original seawater Mg during water-rock interaction, and have no zero-Mg end-member. Therefore, mixing with seawater and estimating a hydrothermal endmember composition cannot easily be determined from normalization to Mg. In addition, Mg can be added to the vent fluids from dissolution of Mg-silicates in this low pH environment^29,30^. As a consequence, fluid compositional data for samples taken from the YV vent fluids in the mixing zone cannot be used for reconstructing temporal trends in fluid endmember composition as they cannot be normalized and, therefore, cannot be corrected for different mixing ratios with ambient seawater that was entrained either in the sub-seafloor or during sampling. However, data for dissolved heavy metals clearly show that the KST vent fluids are a source for many heavy metals. Nevertheless, heavy metal concentrations of ambient seawater surrounding the vents can be interpreted with respect to temporal trends and show a clear decrease. If this decrease is caused by changes in fluid endmember composition or just by reduced input as a consequence of reduced fluid flow cannot be unequivocally discriminated but our observations of a general reduced activity are supporting the latter.

The only exception was Li, which concentration increased over time around KST with several maxima in the water masses including 2018 (Fig. 4). Lithium is readily soluble in acidic solutions and can be derived from weathering and dissolution of hydrothermal precipitates and volcanic ashes. Landslides and tectonic movements created large and fresh surface areas ready for reaction and dissolution and supply of dissolved Li. In contrast, Li in offshore samples and in the vent fluids remained constant.

Strikingly, after the extreme events no more autochthonous native yellow sulfur accretions (cm-sized sulfur balls, sands, chimney structures) could be found at the seafloor that previously covered the seafloor around the vents for at least 3 years during our observations (Video S1). Although we are unclear what has caused the disappearance of the native sulfur, it is plausible that this is a temporary situation following the typhoon when either re-suspended and mass-wasted sediment buried the sulfur or strong waves wiped out the sulfur structures. Another additional reason might be that the sub-seafloor KST plumbing system has shifted in fluid composition as a consequence of blocked pathways for degassing, so that no more native sulfur will be deposited. The abundant native sulfur being historically observed until 2016 reflected more oxidizing conditions where SO_2_-rich fluids predominated over H_2_S-rich fluids^31^. In such oxidizing conditions there is normally a large excess of dissolved sulfur, indicating high H_2_S/metal ratios in the hydrothermal fluids^32^. The lack of native sulfur balls in KST, especially after the disturbance shows that these fluid ratios have changed over time and now decreased to lower H_2_S/metal ratios, with no recovery observed in 2018.

KST benthic communities are comprised by >90% of calcifying organisms, including high-Mg calcite species with a more soluble skeleton (Fig. 6). Despite the strong increase in DIC (thus lower pH/higher CO_2_), organisms seem well adapted to drastic CO_2_ fluctuations via HCO_3_^−^, one to two orders of magnitude higher than predictions for future ocean acidification (OA) (Fig. 4). The measured variability and mixed response in the organisms’ skeleton Mg:Ca and Sr:Ca ratios, including no change in some gastropod species, shows that they mostly reshaped their calcification mechanism after the earthquake and typhoon. This was driven by seawater carbonate changes, dissolved metal fluctuations and even a potential growth rate response following the phytoplankton bloom. The organisms showing no change after the disturbance (mainly gastropods) indicate that the experienced seawater fluctuations are within their tolerance limits, and that thresholds for them can vary at least one order of magnitude. For most organisms, the changes in skeleton Mg:Ca and Sr:Ca ratios did not follow the trend of seawater chemistry fluctuations (Fig. 6). Our findings are similar to experimental incubation studies^33^ which show mixed calcification and elemental ratios in Mg-calcite benthic organisms exposed to gradients of CO_2_ conditions. The KST observations contrast to conclusions from other OA studies on the impact of rising CO_2_ on calcifying organisms that are based on laboratory experiments^12^. Most of these studies demonstrate that there are certain CO_2_ threshold levels for survival and adaptation or strong reductions in calcification and physiological failure^34,35^. A high tolerance of Mg-calcite benthic organisms from shallow waters and the deep-sea to high DIC, CO_2_ and low pH was recently discovered using a field approach^36^. This is in line with our findings that wide fluctuations in seawater carbonate chemistry change skeleton composition but do not compromise survival or tolerance thresholds. KST benthic organisms have evolved under these extreme conditions and, thus, shift skeleton composition accordingly within an extremely wide threshold. Their resilience to large environmental fluctuations yet can validate to a certain extent the ongoing discussion on certain species being winners and losers in a high CO_2_ world^37,38^, as well as the increase of dominance in benthic communities with the subsequent loss of diversity^39^.

The overall effects of major disturbance events such as typhoons, earthquakes, landslides and particle clouds on aquatic communities are poorly studied, but a recent example shows niche overlap in copepod populations, population crashes, and changes in species distribution^40^. At KST, plankton community changes are expected owing to the variations in seawater composition (plankton blooms were seen in our time-series via nutrient depletion and green water-color from unmanned aerial vehicle (UAV) aerial images; Figs 2, 7). We qualitatively surveyed the area via scuba diving before and after the disturbance events (unpublished data from BMBF WTZ cruise report No. 03F0722A from 2015 to 2018) and found no signs of ecosystem collapse or major changes in species composition. The only exception was a reduced crab density down to 30 m potentially linked to the landslides, seabed burial, and sediment deposition. In the East Pacific Ocean, benthic community recovery after a natural underwater volcanic eruption also showed a high level of resilience to disturbance^41^. Dominant species such as vent crabs have high ion-pump regulation capabilities in branchial acid-base regulation which allows them to live closer to the YV^42^. Other species such as scleractinian corals show erosive effects on the aragonite structure despite increasing their skeleton Mg:Ca (no change in Sr:Ca), but can survive^43^. This suggests that corals (e.g., *Tubastraea coccinea*) colonize KST benthic areas with favorable waters masses starting from 6 to 6.5 pH onwards in the peripheral zones, pushing the survival threshold well below the traditional pH 7.9 to 8.1 of most coral reefs elsewhere.

The overall conclusion of this 10-year study is that the catastrophic shifts in seabed morphology, seawater chemistry, vent fluid composition and flow rate, degassing activity, and benthic ecology observed at the KST site profoundly reshaped biogeochemical processes for one year to levels not observed before, and difficult to relate to other vents elsewhere. Yet, the changes remained transient in nature, with a possible recovery of the system within two years. Because the KST shallow vents system has been continuously experiencing the impact of earthquakes or typhoons (e.g., Bilis typhoon in 2000)^2,3,44^ through time^45^, major shifts in biogeochemical processes, including benthic communities or ecological functioning cannot be expected to be permanent.

## Methods

### Hydrographic surveys

KST cruises were organized among German, Taiwanese and Chinese partners with available seawater data in 2000 (not used), 2009, 2010, and 2011 on the Taiwanese/Chinese side, and in 2015, 2016, 2017, and 2018 on the German side (Fig. 1; Appendix S1). The sampling stations and transects remained identical year after year with unified sampling protocols (Fig. 1; Appendix S1). In 2015, 2016, 2017 and 2018, transects were expanded to the sides and back of KST but the venting front area stations remained the same. The YV GPS position did not change until 2016 after the catastrophic events that modified/buried the shoreline and seabed (M5.8 earthquake on 12^th^ May and C5 typhoon Nepartak on 2^nd^–10^th^ July) thus the same vent was sampled in the time-series (Fig. 1). After May 2016, the whole seabed landscape changed thus YV sampling occurred on opportunity in the newly formed vent very close to its original position (Fig. 2; Video S1). All cruises happened either in late spring or early summer taking into account the seasonal effects on the chemistry (temperature and meteoric water influenced). KST transects consisted of both surface and seabed samples taken within defined grids using a Niskin-type bottle attached to a cable and deployed from various vessels ranging from 8 to 14 m (Fig. 1). For each sample, a calibrated YSI multi probe sensor was used to determine pH_total_ (data not further used), temperature and salinity *in situ*. At KST, samples from the YV were obtained by scuba divers using specialized sampling devices closed underwater on the vent mouth and surrounding water masses to trap the fluid with special protection gloves. Divers directly worked on the vent mouth and above the vent, and opened the bottles there in the mixing zone of the VY, on purpose, to get samples in the mouth, above the mouth, and in the water column (all considered mixing zone, not vent fluids). For vent mouth and above vent samples, long-sleeve gloves were used to do the whole procedure and avoid the high temperatures (under 100 °C, mostly in the 60 °C range). The bottles were not rinsed with the sample, and were directly opened in the sampled mixing zones. The bottles were pre-treated in the laboratory to be sterile to minimize sample contamination. The YV sampling was done vertically starting above the vent mouth and mixing zone with a total of 6 sub-stations all the way to the surface to sample the whole buoyant fluid plume (Appendix S1). The YSI multi probe sensor was used to determine pH_total_ (data not further used), temperature and salinity in the vents mouth and above, by taking the probe cable with the divers. Boat stayed at all times on top of the vents to minimize movement of the probe, and facilitate communications. Offshore cruises were also conducted to have open ocean seawater samples as a reference, using the National Taiwan Ocean University (NTOU) vessel belonging to the National Taiwan University. A CTD rosette with 12 Niskin-type bottles and calibrated Seabird sensors was always used in the offshore. In all cruises, dissolved metals (majors and minors) and nutrients were sampled by taking 10 to 30 ml of seawater filtered with a 50 ml sterile syringe using a 0.20 µm sterile filter into 15/50 ml sterile and pre-cleaned polyethylene tubes (tubes had been 10% acid cleaned before sampling dissolved metals). Samples were stored in the fridge in all cases (4 to 8 °C) and sent for analysis to the Institute of Geosciences at Kiel University, Germany (metals) and to GEOMAR, Kiel, Germany (nutrients). Total Alkalinity (TA) and Dissolved Inorganic Carbon (DIC) were directly sampled from the Niskin-type bottle using 250 ml borosilicate flasks (pre-filtered gently through a 0.20 µm sterile filter to remove all particles and bacteria). No HgCl_2_ solution was used to preserve sample because the HgCl_2_ reacts with H_2_S changing the sample to a dark color from precipitation of HgS. Samples were immediately shipped after sampling to the GEOMAR, Kiel, Germany, and were measured within 3 weeks.

### Aerial and underwater work

KST photos were available from 1960 to 2017 while video footage only existed from 2001 to 2017 (Fig. 2; Video S1), when helicopters and Unmanned Aerial Vehicles (UAV) were used to monitor KST activity from a minimum of 100 m above water up to 500 m. UAVs served to understand hydrothermal buoyant and non-buoyant plume dynamics, volcanic activity changes, and to identify vents. From 2015 to 2017 UAVs were used to further survey and detail cliff fractures, landslides, shoreline changes, find freshwater sources, and identify any KST feature relevant to the time-series (Fig. 2; Video S1). Four UAV units were used: a Dji Phantom 3 and 4 Pro+, a Dji Inspire 1, and a Dji Mavic Pro, all sharing air time with a National Geographic photographer in the project. UAVs were always flown in GPS mode, using natural color filter with a video recording mode of either 1080 HD or 4 K. Underwater works were conducted using scuba diving teams with several cameras filming the action (Fig. 2; Video S1). Cameras used were GoPro 2, 3, 4 and 5 always at 1080 HD or 4 K resolution. No photos were directly taken, but they were obtained from video frames. All video editing work was conducted using Sony Vegas 14.0 and Final Cut Pro (Videos S1–S4).

### Seawater analytics

Dissolved metal (majors and minors) samples were analyzed using both inductively coupled plasma – optical emission spectrometry (ICP-OES; SPECTRO Ciros SOP) and ICP-mass spectrometry (ICP-MS; Agilent 7500cs, and Thermo Scientific Element XR) at the Institute of Geosciences, Kiel University, Kiel, Germany (Appendix 2). All samples were analyzed in triplicate, and 10% of all samples were re-analyzed as sample replicates for estimating analytical uncertainty. ICP-OES results for seawater were normalized to IAPSO seawater standard. Accuracy was monitored by international certified reference materials NASS-5, CASS-4, NIST 1640a, and LGC 6019. Dissolved inorganic nitrate (DIN, N), dissolved inorganic phosphate (DIP, P), and dissolved silicon (Si) were determined according to^46^ using a HITACHI U-2000 spectrophotometer at the GEOMAR, Kiel, Germany. The precisions for DIN and DIP were ±0.1 μM and ±0.02 μM, respectively. The seawater carbonate system parameters were calculated from temperature, salinity, and the concentration of DIC, TA and phosphate using the software CO2SYS^47^. TA and DIC were determined in triplicates and some samples were measured 5 times, especially for YV fluid samples that owing to the high DIC and low TA values had the largest variability. A Versatile Instrument for the Determination of Titration Alkalinity (VINDTA) was used to measure TA/DIC samples except the YV DIC ones owing to the high sulfur concentrations, which were measured using an Apollo SciTech DIC analyzer (AS-C3). The instrument was calibrated with Certified Reference Material (CRM) for oceanic CO_2_ measurements, Marine Physics Laboratory of Scripps Institute of Oceanography, University of San Diego, following^48^. Precisions for TA and DIC were ±7.45 μmol kg^−1^ and ±4.7 μmol kg^−1^, respectively.

### Biological samples collection

Benthic organisms (megafauna) were collected from the YV and WV area during 2015 (May) and 2017 (July) cruises at the same GPS coordinates (YV: 24.835987°N, 121.963403°E; WV: 24.834765°N, 121.961493°E), using scuba diving equipment. On the YV, crabs (*Xenograpsus testudinatus*) were collected in various habitats within maximum 10 m from the vent fluid source from inside small caves and holes, feeding from the biofilms attached to the vent rocks, or freely walking on the sediments. On the WV, sessile organisms (mainly gastropods and corals) were collected around the rocks near small bubbling fumaroles. During diving, all organisms were placed in portable nets and brought immediately to the surface where they were introduced in zip-lock plastic bags to be taken to the laboratory. In order to sample exactly the same seawater mass the organisms lived in, divers took air-filled 1 L bottles that were opened/closed near to the organisms at the same time of collection. Seawater samples were then processed on-board ship for nutrients, TA, DIC and major metals by filtering all volumes with a 50 ml sterile syringe using a 0.20 µm sterile filter into 15/50 a ml sterile polyethylene tubes (tubes for samples for trace metal analysis had been 10% acid cleaned) and 250 ml borosilicate bottles, respectively. Samples were stored in the fridge in the dissolved metal cases (4 to 8 °C), and at room temperature for TA/DIC, and sent for analysis to the Institute of Geosciences, Kiel University, Germany (major metals) and the GEOMAR, Kiel, Germany (TA/DIC and nutrients).

### Skeleton carbonate preparation and analytics

We compared the major metal concentration changes in the organism skeleton carbonate before and after the earthquake and typhoon in 2016. Skeleton carbonate of between 3 and 14 specimens from each organism (total *n* = 7) collected in 2015 and 2017 (Table S1) were processed in the laboratory upon arrival to port by drying for 48 h at 60 °C in a constant-temperature oven. Once dried, organisms were shipped to the Ifg in CAU. Here, dried organisms’ samples were handled separating the skeleton carbonate component from the organic tissues manually with a tissue’s forceps. The skeleton carbonate was reduced to a fine powder using a Planetary Micro Mill PULVERISETTE 7 with metal cups filled with 0.5 cm balls that upon rotation milled down the samples. The powder was then poured into clean A4 paper sheets to target the purest possible white carbonate as a first step to favor the organic cleaning process. Between 550 and 730 µg of powder were weighed into Eppendorf vials for chemical cleaning following a modified foraminifera protocol to remove clay particles and organic matter^49,50^ without the reductive step. Since the cleaning protocol is designed for foraminiferal shell fragments, we did not perform the weak acid leach to reduce the risk of material loss due to the relatively large surface area of the carbonate powder samples. In short, the samples were rinsed three times with ultrapure water (60 seconds ultrasound), three times with methanol (20 seconds ultrasound) to remove clay minerals, and again three times with ultrapure water. Removal of organic matter was carried out with NaOH/H_2_O_2_ in a hot water bath for 10 minutes, while the samples were agitated by flipping the sample rack and ultrasound for 15 seconds. The sample material was again rinsed with ultrapure water three times and transferred into new acid-leached vials. After dissolution in 500 µl 0.1 N ultrapure NHO_3_ and centrifuging to remove any remaining solid phases, 400 µl of the supernatant was diluted targeting a concentration of ~ 40 ppm Ca and measured with a SPECTRO Ciros^CCD^ ICP-OES at the ICP-MS Laboratory, Institute of Geosciences, CAU. Accuracy of the data was checked by analyzing the carbonate reference standard ECRM 752–1 as well as different concentration gradients of JCp-1 (1:50, 1:100). The external analytical error was ~0.1%.

### Data treatment and graphics

The final databases are deposited at the NOAA National Center for Environmental Information (NCEI) under Accession Number 0175781 in https://data.nodc.noaa.gov/cgi-bin/iso?id=gov.noaa.nodc:0175781 with DOI: 10.25921/6hy3-6d56. All analyses and graphical work was performed in Statistica 13.0 (StatSoft), SigmaPlot 12.0 (Systat Software Inc.), SURFER (Golden Software, LLC.), and Corel Draw X7 (Corel Corp.).

## Supplementary information

Appendix S1

Appendix S2

Supplementary Information
