# Supplementary material for: Earthquake and typhoon trigger unprecedented transient shifts in shallow hydrothermal vents biogeochemistry
Source: Sci Rep. 2019 Nov 15;9:16926. doi: 10.1038/s41598-019-53314-y (PMC6858458; doi:10.1038/s41598-019-53314-y)
Supplement: Supplementary file 3 — Supplementary Information [file 41598_2019_53314_MOESM3_ESM.docx]

**Earthquake and typhoon trigger unprecedented transient shifts in shallow hydrothermal vents biogeochemistry**

Mario Lebrato^1,2,*^, Yiming V. Wang^1,3^, Li-Chun Tseng^4^, Eric P. Achterberg^5^, Xue-Gang Chen^6^, Juan-Carlos Molinero^1,7^, Karen Bremer^1^, Ulrike Westernströer^1^, Emanuel Söding^1^, Hans-Uwe Dahms^8^, Marie Küter^1^, Verena Heinath^1^, Janika Jöhnck^1^, Kostas I. Konstantinou^9^, Yiing J. Yang^10^, Jiang-Shiou Hwang^4^ and Dieter Garbe-Schönberg^1,11^

*^1^ Institute of Geosciences, Kiel University (CAU), Kiel, Germany*

*^2^ Bazaruto Center for Scientific Studies (BCSS), Benguerra Island, Mozambique*

*^3^ Max Planck Institute for the Science of Human History, Jena, Germany*

*^4^ National Taiwan Ocean University, Keelung City, Taiwan*

*^5^ GEOMAR Helmholtz Centre for Ocean Research Kiel, Kiel, Germany*

*^6^ Ocean College, Zhejiang University, Zhoushan City, China*

*^7^ Marine Biodiversity, Exploitation and Conservation (MARBEC), IRD/CNRS/IFREMER/University of Montpellier, Montpellier, France*

*^8^ Kaohsiung Medical University, Kaohsiung, Taiwan*

*^9^ National Central University, Taoyuan, Taiwan*

*^10^ National Taiwan University, Taipei City, Taiwan*

*^11^ Jacobs University Bremen gGmbH, Bremen, Germany*

**Key words:** earthquake, typhoon, impact, biogeochemistry, shallow hydrothermal vents, marine chemistry

*^*^Corresponding Author*

Kiel University (CAU), Institute of Geosciences, Ludewig-Meyn-Strasse 10, 24118, Kiel, Germany

*E-mail:* mlebrato13@gmail.com

*Telephone:* +34 610 67 77 42

**Supplementary information**

**Video S1. Turtle Island shallow vents modern history changes.** Observed changes and processes using helicopter and drone aerial video/photo, assisted with underwater filming from 1960 to 2017 around Turtle Island shallow vents. Presented are the main discoveries and processes, especially after the large 2016 M5.8 earthquake and C5 typhoon that changed venting activity.

Watch: <https://www.youtube.com/watch?v=us6hIY5MqGU>

**Video S2. Turtle Island live earthquake video recorded by Taiwan TV.** Real-time earthquake tremors in the vents area causing landslides on the cliff face as broadcasted in Breaking News on Taiwan national TV during Summer 2017.

Watch: <https://www.youtube.com/watch?v=DI-1-t7peBI>

**Video S3. NTU1 and NTU2 buoy data before and after typhoon Nepartak.** Real-time changes

in wind speed, seawater pressure, atmospheric pressure, and seawater temperature data before and after the typhoon on NTU1 and NTU2 buoys. Images are courtesy of the Institute of Oceanography at the National Taiwan University (<https://po.oc.ntu.edu.tw/buoy/buoy2017/index.php>).

Watch: <https://www.youtube.com/watch?v=uLFYCB9ikcg>

**Video S4. NTU2 buoy camera during and after typhoon Nepartak.** Real-time offshore typhoon rough ocean conditions, captured as snapshots from NTU2 buoy camera during July 2016 and a comparison with August 2016. Images are courtesy of the Institute of Oceanography at the National Taiwan University (<https://po.oc.ntu.edu.tw/buoy/buoy2017/index.php>).

Watch: <https://www.youtube.com/watch?v=bBKEqbTMPcA>

**Table S1. Benthic organisms’ skeleton calcite chemistry statistics.** White Vent (WV) and Yellow Vent (YV) organisms skeleton calcite Mg:Ca and Sr:Ca ratios and the t-test statistics before and after the M5.8 earthquake and C5 typhoon (see Figure 6).

|  | **BEFORE**  (May 2015) | | |  | **AFTER**  (July 2017) | | |  |
| --- | --- | --- | --- | --- | --- | --- | --- | --- |
| **Species** | **Mg:Ca (mmol:mol)** | **SD** | ***n*** |  | **Mg:Ca (mmol:mol)** | **SD** | ***n*** | **STATS** |
| *Anachis miser* (WV) | 6.74 | 1.19 | 3 |  | 2.20 | 0.21 | 6 | ***F*_2_ = 6.58, *P*<0.05** |
| *Bostrycapulus gravispinosus* (WV) | 1.93 | 0.05 | 4 |  | 2.87 | 0.74 | 5 | ***F*_4_ = 2.86, *P* <0.05** |
| *Ergalatax contracta* (WV) | 1.14 | 0.41 | 3 |  | 2.80 | 0.69 | 6 | ***F*_6_ = 4.52, *P* <0.05** |
| *Reishia luteostoma* (WV) | 1.50 | 0.30 | 3 |  | 1.99 | 0.36 | 5 | *F*_5_ = 2.07, *P*>0.05 |
| *Serpulorbis sp.* (WV) | 1.40 | 0.35 | 6 |  | 2.85 | 0.05 | 3 | ***F*_5_ = 10.05, *P* <0.05** |
| *Tubastrea coccinea* (WV) | 4.00 | 0.16 | 5 |  | 7.37 | 1.34 | 6 | ***F*_5_ = 6.08, *P* <0.05** |
| *Xenograpsus testudinatus* (YV) | 130.03 | 10.78 | 14 |  | 117.86 | 2.54 | 5 | ***F*_16_ = 3.93, *P* <0.05** |
|  | **Sr:Ca (µmol:mol)** | **SD** | ***n*** |  | **Sr:Ca (µmol:mol)** | **SD** | ***n*** | **STATS** |
| *Anachis miser* (WV) | 1.98 | 0.03 | 3 |  | 1.74 | 0.02 | 6 | ***F*_2_ = 12.14, *P* <0.05** |
| *Bostrycapulus gravispinosus* (WV) | 1.78 | 0.07 | 4 |  | 1.92 | 0.06 | 5 | ***F*_6_ = 3.19, *P* <0.05** |
| *Ergalatax contracta* (WV) | 1.66 | 0.23 | 3 |  | 1.66 | 0.04 | 6 | *F*_2_ = 0.04, *P*>0.05 |
| *Reishia luteostoma* (WV) | 2.59 | 0.28 | 3 |  | 1.85 | 0.11 | 5 | ***F*_3_ = 7.23, *P* <0.05** |
| *Serpulorbis sp.* (WV) | 1.67 | 0.09 | 3 |  | 1.76 | 0.15 | 5 | *F*_2_ = 0.92, *P*>0.05 |
| *Tubastrea coccinea* (WV) | 9.18 | 0.09 | 5 |  | 8.97 | 0.14 | 4 | *F*_4_ = 2.69, *P*>0.05 |
| *Xenograpsus testudinatus* (YV) | 5.67 | 0.25 | 14 |  | 5.43 | 0.10 | 5 | ***F*_16_ = 2.94, *P* <0.05** |

**Table S2. White Vent (WV) and Yellow Vent (YV) environmental conditions.** WV and YV seawater meta-data and chemistry before and after the M5.8 earthquake and C5 typhoon (see Figure 6),

| **Location** | **Depth** | **Event** | **Year** | **Month** | **Temp.** | **Salinity** | **Phosphate** | **Nitrate** | **DIC** | **TA** | **Mg** | **Ca** | **Sr** |
| --- | --- | --- | --- | --- | --- | --- | --- | --- | --- | --- | --- | --- | --- |
|  | m |  |  |  | (°C) |  | μmol L^-1^ | μmol L^-1^ | μmol kg^-1^ | μmol kg^-1^ | mM | mM | µM |
|  |  |  |  |  |  |  |  |  |  |  |  |  |  |
| Yellow Vent | 7.50 | Before | 2015 | May | 28.05 | 33.70 |  |  | 2241.23 | 2086.37 | 51.05 | 9.95 | 83.19 |
| Yellow Vent | 7.50 | Before | 2015 | May | 38.50 | 32.81 |  |  | 2615.51 | 1986.37 | 52.58 | 10.14 | 84.59 |
| Yellow Vent | 7.50 | Before | 2015 | May | 24.78 | 33.12 | 0.24 | 2.31 | 2549.02 | 1990.07 |  |  |  |
| Yellow Vent | 7.50 | After | 2017 | July | 28.25 | 33.73 | 0.09 | 1.31 | 2818.14 | 1942.85 | 47.93 | 9.39 | 77.95 |
| Yellow Vent | 7.50 | After | 2017 | July | 28.92 | 33.29 | 0.10 | 0.88 | 2904.53 | 1458.88 | 47.97 | 9.55 | 78.53 |
|  |  |  |  |  |  |  |  |  |  |  |  |  |  |
| White Vent | 14.00 | Before | 2015 | May | 20.750 | 33.81 | 0.21 | 4.68 | 2339.38 | 2182.76 | 53.04 | 10.26 | 85.41 |
| White Vent | 14.00 | Before | 2015 | May | 20.840 | 33.72 | 0.21 | 3.77 | 2147.57 | 2187.86 | 54.72 | 10.64 | 87.75 |
| White Vent | 14.00 | Before | 2015 | May | 21.070 | 33.91 | 0.21 | 6.21 | 2223.03 | 2184.16 | 54.37 | 10.41 | 86.76 |
| White Vent | 14.00 | Before | 2016 | May | 21.520 | 33.62 | 0.09 | 0.26 | 2214.86 | 2029.52 | 52.46 | 10.25 | 83.80 |
| White Vent | 14.00 | Before | 2016 | May | 22.170 | 33.80 | 0.08 | 3.77 | 2169.76 | 2185.61 | 53.04 | 10.26 | 85.41 |
| White Vent | 14.00 | Before | 2016 | May | 22.770 | 33.10 | 0.10 | 6.04 | 2212.32 | 2182.06 | 56.67 | 10.87 | 89.60 |
| White Vent | 14.00 | After | 2017 | July | 25.470 | 33.56 | 0.05 | 0.83 | 2482.45 | 1751.98 | 49.32 | 9.66 | 79.34 |
| White Vent | 14.00 | After | 2017 | July | 26.840 | 33.67 | 0.09 | 1.27 | 2553.77 | 1676.80 | 49.52 | 9.67 | 79.92 |
| White Vent | 14.00 | After | 2017 | July | 24.200 | 33.23 | 0.07 | 1.25 | 2970.32 | 1560.48 | 48.66 | 9.54 | 78.65 |
| White Vent | 14.00 | After | 2017 | July | 25.470 | 33.56 | 0.05 | 0.83 | 2482.45 | 1751.98 | 49.32 | 9.66 | 79.34 |
| White Vent | 14.00 | After | 2017 | July | 26.840 | 33.67 | 0.09 | 1.27 | 2553.77 | 1676.80 | 49.52 | 9.67 | 79.92 |
| White Vent | 14.00 | After | 2017 | July | 24.200 | 33.23 | 0.07 | 1.25 | 2970.32 | 1560.48 | 48.66 | 9.54 | 78.65 |

**Figure S1. Nepartak typhoon satellite and buoy data.** Full details of Nepartak wind speed + direction, and significant wave height + direction before it hit Turtle Island and mainland Taiwan. NTU1 and NTU2 buoys data provide insights into the typhoon strength a few kilometers before hitting Turtle Island. The accumulated precipitation shows the sudden meteoric water reaching Turtle Island freshwater systems behind the vents area in the island front and cliffs. There are no in-situ Turtle Island rain measurements, but being very close to Yilan, between 100 and 300 mm of rain potentially accumulated in the island. Buoy data, maps and images are courtesy of the Institute of Oceanography at the National Taiwan University and were prepared for this manuscript by co-author Y.J. Yang (<https://po.oc.ntu.edu.tw/buoy/buoy2017/index.php>). Colored maps of wind speed, significant wave height, and accumulated rain are courtesy of the Central Weather Bureau (<http://www.cwb.gov.tw/eng/>), which maps and data are publicly available under <https://www.cwb.gov.tw/V7e/observe/rainfall/hk.htm> with an open information disclaimer under <https://www.cwb.gov.tw/V7e/information.htm>. All imagery and diagrams were individually obtained, and the final figure was generated using Corel Draw X7 (Corel Corp.).


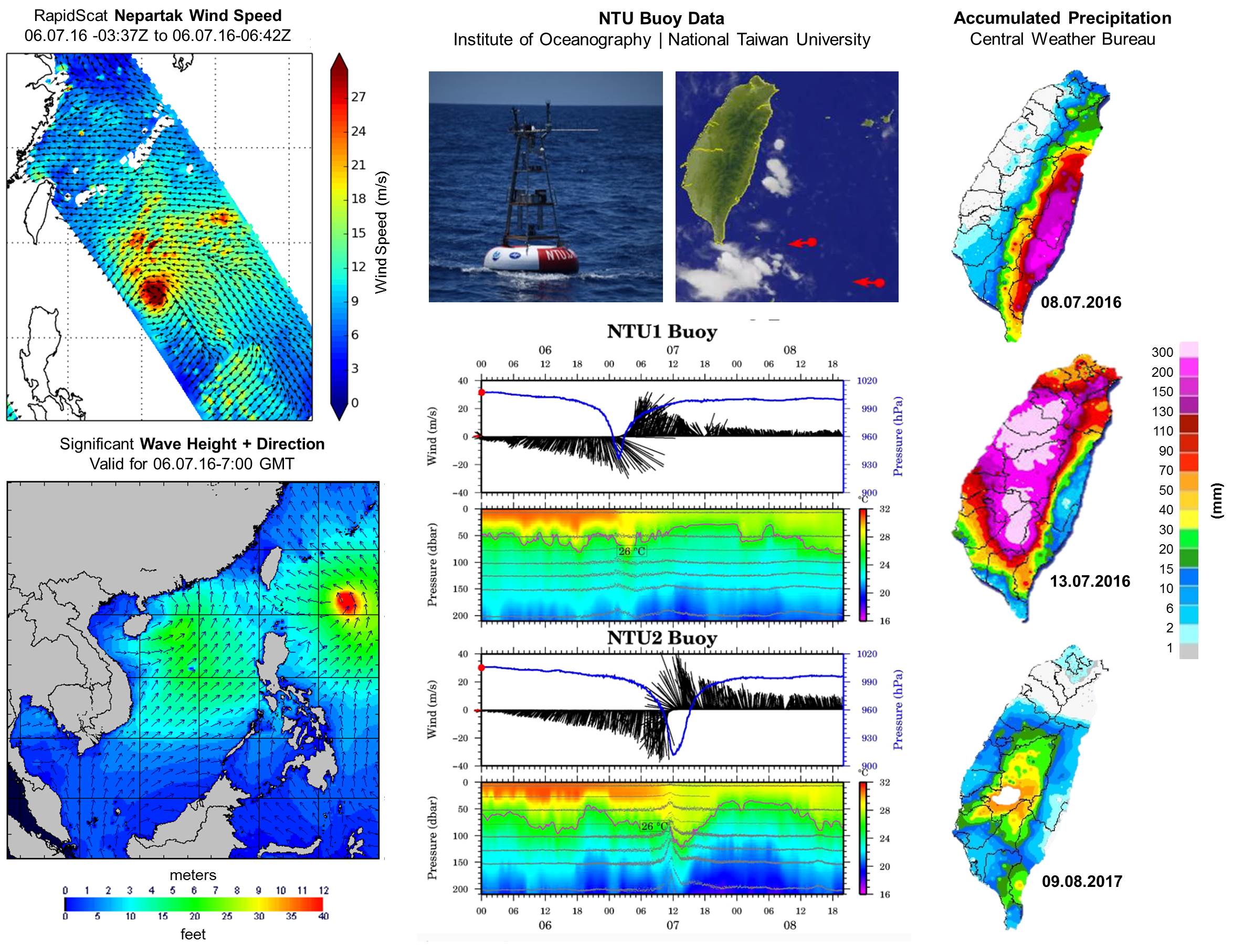


**Figure S2. Seawater dissolved elements time-series before and after the catastrophic events.** Detailed seawater dissolved elements (majors and minors) from 2010 to 2018 around Turtle Island and the vents, including offshore waters using gridded stations (all parameters measured 1 m below surface). Comparable seawater data before 2010 are not available, thus not included in the time-series, in contrast to the photos of Figure 2. The complete dataset of all chemistry, measurements, metadata, and seawater major/minor elements is included in Appendix S1. This figure is the extension from Figure 4.


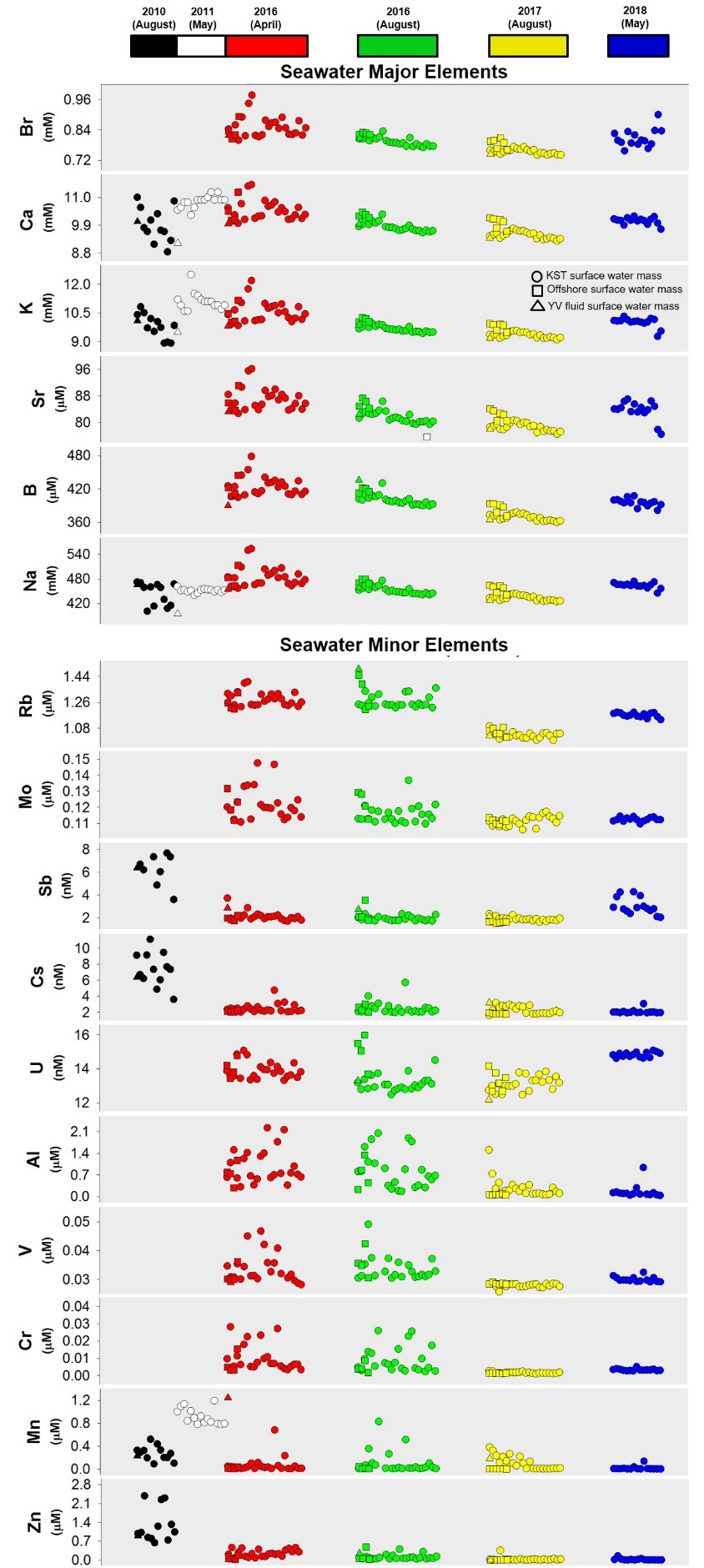


**Figure S3. YV vertical seawater chemistry and dissolved elements composition time-series.** Detailed vertical structure of the YV seawater carbonate chemistry and dissolved elements (majors and minors) from 2009 to 2017. For each parameter the full time-series data are presented in the same vertical plot to see individual changes over time. Comparable YV vertical seawater data before 2009 are not available, thus not included in the time-series, in contrast to the photos of Figure 1 and 2. The complete dataset of all chemistry, measurements, metadata, and seawater major/minor elements is included in Appendix S1. This figure is the extension from Figure 5.


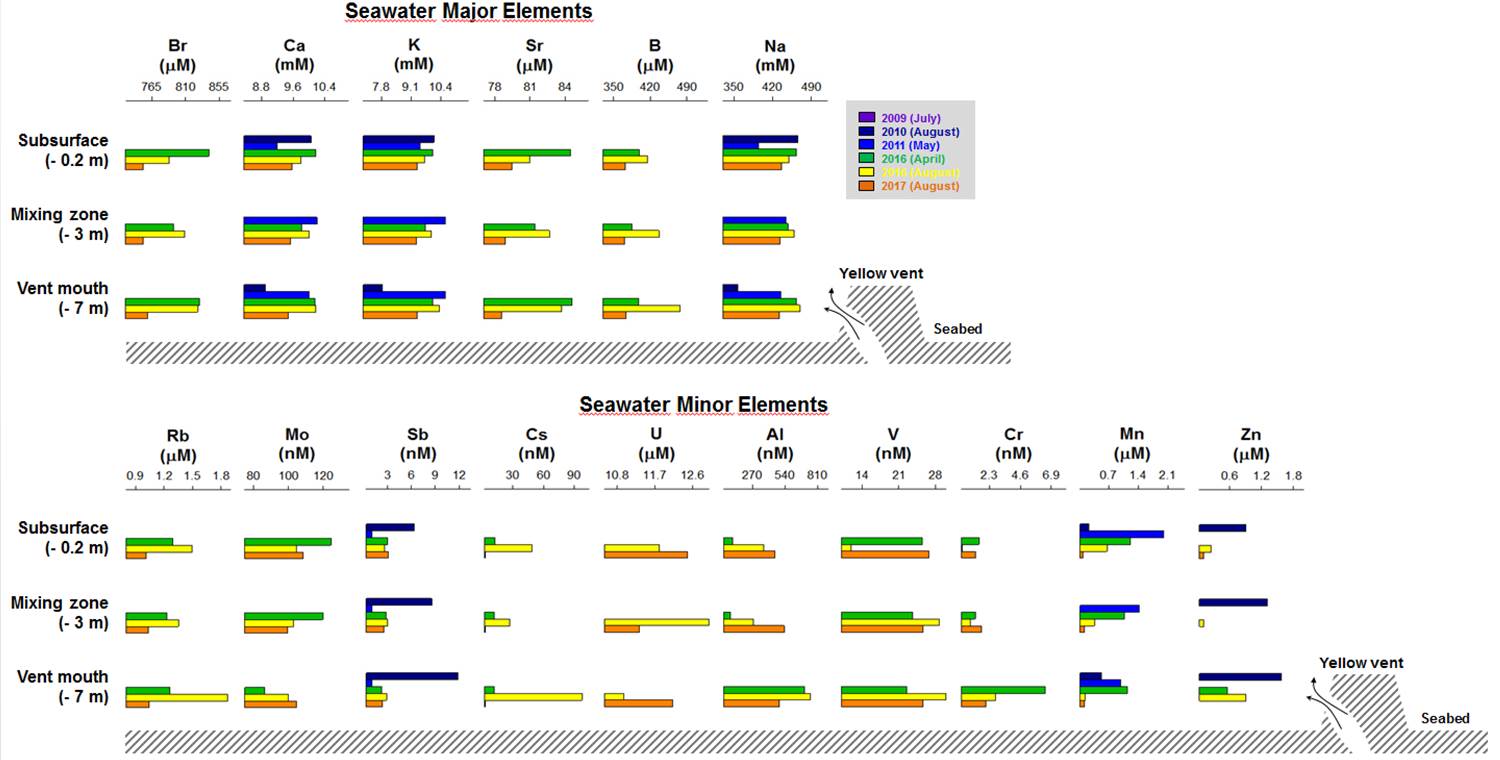


**Appendix S1. Turtle Island time-series database.** Raw seawater carbonate chemistry, dissolved elements (majors and minors)**,** and environmental metadata collected from 2010 to 2017 in Turtle Island and used in this time-series study.

**Appendix S2. ICP-OES and ICP-MS quality control data and measurements replication.** Details on the ICP-OES laboratory quality and replicability that guarantees the high precision results presented in this study.
